# Exploring potential mechanisms of Suhexiang Pill against COVID-19 based on network pharmacology and molecular docking

**DOI:** 10.1097/MD.0000000000027112

**Published:** 2021-12-23

**Authors:** Jialin Li, Zhihong Huang, Shan Lu, Hua Luo, Yingying Tan, Peizhi Ye, Xinkui Liu, Zhishan Wu, Chao Wu, Antony Stalin, Haojia Wang, Yingying Liu, Liangliang Shen, Xiaotian Fan, Bei Zhang, Jianping Yi, Lu Yao, Yi Xu, Jiarui Wu, Xianchun Duan

**Affiliations:** a Department of Clinical Chinese Pharmacy, School of Chinese Materia Medica, Beijing University of Chinese Medicine, Beijing, China; b Institute of Chinese Medical Sciences, State Key Laboratory of Quality Research in Chinese Medicine, University of Macau, Macao, China; c Chinese Medicine Department of the Cancer Hospital of the Chinese Academy of Medical Sciences and Peking Union Medical College, Beijing, China; d Beijing Zhongyan Tong Ren Tang Pharmaceutical R&d Co. LTD, Beijing, China.; e Department of Pharmacy, The First Affiliated Hospital of Anhui University of Chinese Medicine, No. 117, Meishan Road, Shushan District, Hefei City, Anhui Province, PR China; f State Key Laboratory of Subtropical Silviculture, Department of Traditional Chinese Medicine, Zhejiang A&F University, Hangzhou, China

**Keywords:** coronavirus disease 2019, molecular docking, molecular mechanism, network pharmacology, suhexiang pill

## Abstract

**Background:**

The traditional Chinese medicine prescription Suhexiang Pill (SHXP), a classic prescription for the treatment of plague, has been recommended in the 2019 Guideline for coronavirus disease 2019 (COVID-19) diagnosis and treatment of a severe type of COVID-19. However, the bioactive compounds and underlying mechanisms of SHXP for COVID-19 prevention and treatment have not yet been elucidated. This study investigates the mechanisms of SHXP in the treatment of COVID-19 based on network pharmacology and molecular docking.

**Methods:**

First, the bioactive ingredients and corresponding target genes of the SHXP were screened from the traditional Chinese medicine systems pharmacology database and analysis platform database. Then, we compiled COVID-19 disease targets from the GeneCards gene database and literature search. Subsequently, we constructed the core compound-target network, the protein-protein interaction network of the intersection of compound targets and disease targets, the drug-core compound-hub gene-pathway network, module analysis, and hub gene search by the Cytoscape software. The Metascape database and R language software were applied to analyze gene ontology biological processes and Kyoto Encyclopedia of Genes and Genomes (KEGG) pathway enrichment. Finally, AutoDock software was used for molecular docking of hub genes and core compounds.

**Results:**

A total of 326 compounds, 2450 target genes of SHXP, and 251 genes related to COVID-19 were collected, among which there were 6 hub genes of SHXP associated with the treatment of COVID-19, namely interleukin 6, interleukin 10, vascular endothelial growth factor A, signal transducer and activator of transcription 3 (STAT3), tumor necrosis factor (TNF), and epidermal growth factor. Functional enrichment analysis suggested that the effect of SHXP against COVID-19 is mediated by synergistic regulation of several biological signaling pathways, including Janus kinase/ STAT3, phosphatidylinositol 3-kinase (PI3K)-protein kinase B (Akt), T cell receptor, TNF, Nuclear factor kappa-B, **Toll-like receptor, interleukin 17, Chemokine, and hypoxia-inducible factor 1 signaling pathways.** SHXP may play a vital role in the treatment of COVID-19 by suppressing the inflammatory storm, regulating immune function, and resisting viral invasion. Furthermore, the molecular docking results showed an excellent binding affinity between the core compounds and the hub genes.

**Conclusion:**

This study preliminarily predicted the potential therapeutic targets, signaling pathways, and molecular mechanisms of SHXP in the treatment of severe COVID-19, which include the moderate immune system, relieves the “cytokine storm,” and anti-viral entry into cells.

## 1 Introduction

Currently, coronavirus disease 2019 (COVID-19), caused by infection with severe acute respiratory syndrome coronaviruses 2 (SARS-CoV-2), is spreading worldwide. The clinical features of COVID-19 are diverse and range from asymptomatic to severe and critical type. Severe and critical cases represented 14% and 5% of laboratory-confirmed COVID-19 patients, leading to a large proportion of deaths.^[1]^ Severe patients show signs of dyspnea and acute respiratory distress syndrome (ARDS) may occur in critical patients.^[2]^

In the struggle against diseases for thousands of years, traditional Chinese medicine (TCM) has accumulated rich experience and formulated many effective prescriptions, which provide a solid foundation for the application of TCM theories and methods to the treatment of COVID-19.^[3–5]^ The focus and difficulty in the treatment of COVID-19 are the severe type and critical type, as they are the main factors affecting the mortality rate. The current research data showed that various TCM products and their combinations are effective treatment options for COVID-19.^[6]^ In the clinically severely ill patients, the intervention of TCM or the combination of Chinese and Western medicine contributes to the relief of the disease and the reduction of mortality.^[7,8]^

Suhexiang pill (SHXP) was first recorded in the prescription of *Preions of the Bureau of Taiping People's Welfare Pharmacy* (Taiping Huimin Heji Ju Fang) of the Song Dynasty, and Shen Kuo gave a high evaluation on its treatment of epidemic diseases in *the Dream Rivulet Diary* (Mengxi Bitan).^[9]^ The National Health Commission of China Diagnosis and Treatment Protocol for COVID-19 infection recommends SHXP to treat critically ill patients with COVID-19.^[10]^ SHXP, which promotes qi to relieve pain and aromatic resuscitation, consist of fifteen common Chinese herbal medicines. In the formula, *Liquidambar orientalis* Mill. (Suhexiang) and *Styrax tonkinensis* (Pierre) Craib ex Hart. (Anxixiang) are good at clearing filth, dispelling turbidity, and inducing resuscitation. *Moschus moschiferus* Linnaeus (Shexiang) and *Dryobalanops aromatica* Gaertn. f. (Bingpian) are adept at inducing resuscitation and removing blockages of the whole body. The above four herbal medicines are the “Monarch” medicines in the prescription. *Cyperus rotundus* L. (Xiangfu), *Eugenia caryophyllata* Thunb. (Dingxiang), *Aucklandia lappa* Decne. (Muxiang), *Aquilaria Sinensis* (Lour.) Gilg. (Chenxiang), *Santalum album* L.(Tanxiang), *Boswellia carterii* Birdw. (Ruxiang) can regulate qi and blood, warm up the meridians, fall qi inversion. They are regarded as the “Minister” medicine in the prescription. *Piper longum* L. (Biba) is suitable for warming the spleen and stomach for dispelling cold; *Bubalus bubalis* Linnaeus (concentrate powder) (Shuiniujiao) has the effect of clearing the heat and detoxifying; *Cinnamomum camphora* (L.). Presl. (Zhusha) soothe the nerves; *Atractylodes macrocephala* Koidz. (Baizhu) can invigorate the spleen and stomach and dry up dampness; *Terminalia chebula* Retz. var. *tomentella* Kurt. (Hezi) inhibits qi and prevents the spicy fragrance from being too diffused and dissipating. They can be called as “Assistant” medicines. The combined use of various drugs can strengthen resuscitation and qi movement to relieve pain and prevent gas consumption damage and have a therapeutic effect on COVID-19.

**Figure 1:**
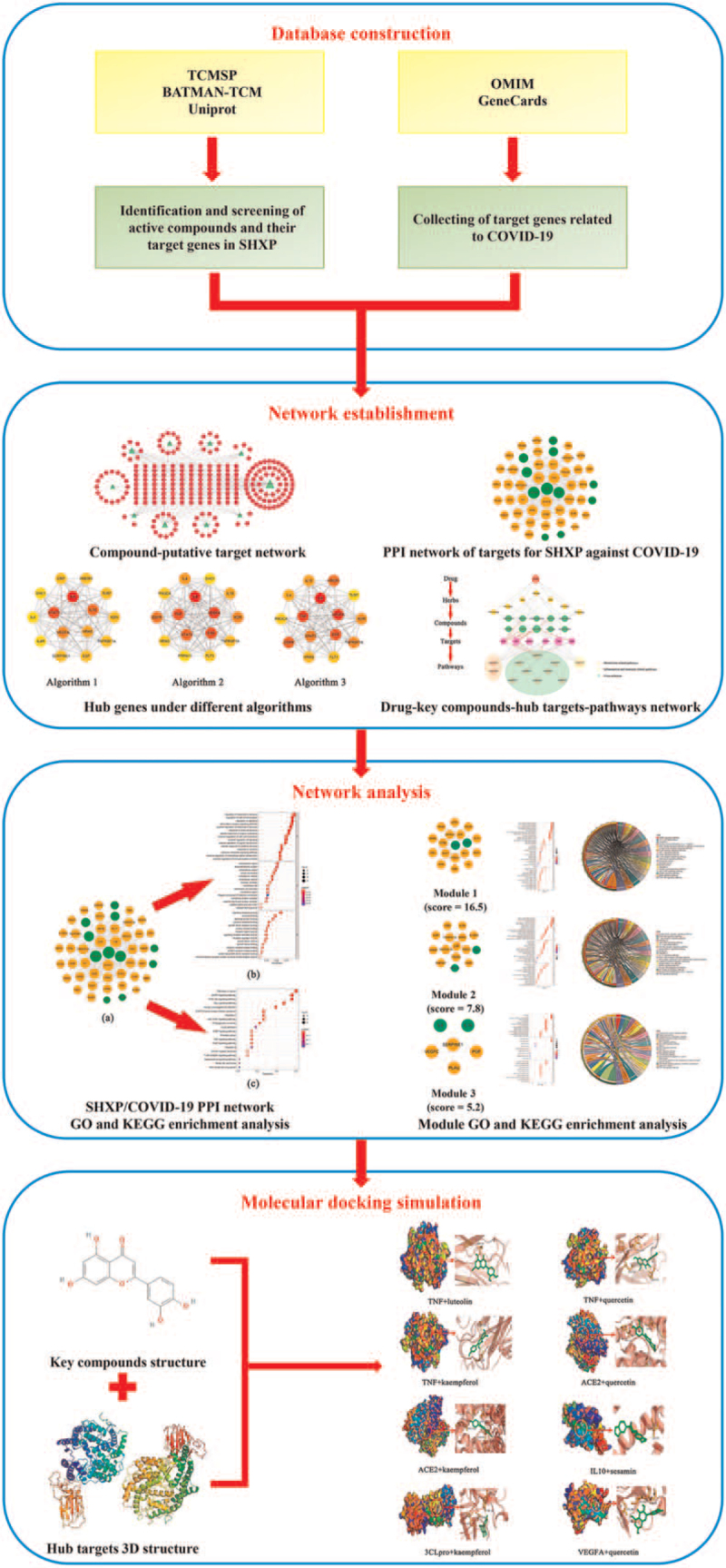
Workflow diagram of the network pharmacology-based analysis of SHXP in the treatment of COVID-19.

Network pharmacology can reflect and clarify the interactive relationship between multiple compounds, multiple targets, and multiple pathways, which coincides with the overall concept of TCM. Meanwhile, it abstracts the relationship into a network model and illustrates the effect of drugs on human biological network from a systematic perspective.^[11–13]^ Molecular docking is an important research technology that can quickly and accurately predict the conformation of the binding of the receptor-drug complex and predict the binding pattern and affinity to support the screening of active components.^[14–16]^ Therefore, we chose the network pharmacology and molecular docking approaches to explore the mechanisms of SHXP in the treatment of COVID-19. The corresponding workflow is shown in Figure 1.

## 2 Methods

### 2.1 Identification and screening of active compounds and their target genes

We screened active ingredients of SHXP by searching the pharmacological database of the Traditional Chinese Medicine System and Analyzing Platforms (TCMSP, http://tcmspw.com/tcmsp.php) and Bioinformatics Analysis Tool for Molecular mechANis -m of Traditional Chinese Medicine, (BATMAN-TCM, http://tcmspw.com/tcmsp.php). Oral bioavailability > 30% and drug-like(drug similarity)>0.18 were used as screening thresholds to filter compounds. Meanwhile, potential target proteins were collected for these active ingredients in the above 2 databases.^[17,18]^ UniProt database (http://www.uniprot.org/) was used to convert protein names into gene names.

### 2.2 Construction of the compound-target network

The deduplicated compounds and their relevant targets were imported into the Cytoscape 3.7.2 software (http://www.cytoscape.org/) to construct a compound-predicted target network map. Cytoscape is an open-source bioinformatics analysis software for constructing molecular interaction networks composed of proteins, genes, drugs, and other interactions for visual browsing and analysis.^[19,20]^

### 2.3 COVID-19 target genes

The GeneCards (https://www.genecards.org) database provides gene-centric information that is automatically mined and integrated from myriad data sources. The Online Mendelian Inheritance in Man (OMIM, https://omim.org/) database is a comprehensive, authoritative, and timely knowledgebase of human genes and genetic disorders compiled to support human genetics research, education, and the practice of clinical genetics.^[21–23]^ We used these 2 databases to mine genes related to COVID-19 by searching for the keywords “novel coronavirus pneumonia” “COVID-19” and then setting up a data set for COVID-19 targets.

### 2.4 Intersection of disease target genes and compound target genes

The target genes predicted from the active ingredients in SHXP were intersected and mapped with the target genes predicted for the COVID-19 disease to obtain the potential therapeutic targets of SHXP to treat COVID-19. The Venn Diagram package in R was used to draw a Venn diagram.^[24]^

### 2.5 Constructing a protein-protein interactions network of intersection genes and screening hub genes

PPI data of potential therapeutic targets were obtained from the Search Tool for the Retrieval of Interacting Genes database (STRING, https://string-db.org), which provides information on predictive and experimental protein interactions. The prediction method of this database is derived from the experiments, databases, and text mining of neighborhood, gene fusion, co-occurrence and co-expression. In addition, the database defines protein–protein interactions (PPIs) with confidence ranges for data scores (low confidence >0.15; medium confidence >0.4; high confidence >0.7). In this study, PPIs with combined scores >0.7 were reserved for further research. Then, we used the plugin CytoHubba in Cytoscape software to analyze the data in the PPIs network of overlapping targets with 3 algorithms “Closeness, Betweenness, BottleNeck,” aiming to find hub genes accurately. The parameters were set as top = 15 and were ranked by the 3 algorithms. Betweenness describes the number of shortest paths between pairs of nodes passing through the node; closeness represents the inverse of the sum of distances from nodes to other nodes; bottleneck genes are considered as highly central proteins connecting multiple complexes that are more likely to be part of signal transduction pathways. The higher the 3 quantitative values of a node are, the greater its importance in the network.^[25–29]^

### 2.6 Gene ontology and kyoto encyclopedia of genes and genomes enrichment analysis

Metascape (http://Metascape.org), a biological information annotation database, is a web tool used for gene function annotation that combines more than 40 independent knowledge bases, including functional enrichment analysis, interactive analysis, gene annotation, and member search. It provides a comprehensive gene list annotation and analysis resources, which can help users apply the currently popular bioinformatics analysis methods to batch gene and protein analysis to realize the cognition of gene or protein function. Potential therapeutic targets were imported into the Matescape database as a gene list. The minimum gene overlap (Min Overlap) was set to 3, the *P* value was less than .01, and the minimum enrichment factor (Min Enrichment) was set to 1.5. GO enrichment analysis illustrates the role of target proteins of TCM compounds in gene function, including 3 modules: biological processes, molecular functions, and cellular components. KEGG pathway enrichment analysis provides the functional annotations of pathways of a given gene set and pathways enrichment analysis. Finally, GO and KEGG enrichment analyses were performed for the protein of the combined protein in the network construction.^[30–32]^

### 2.7 Module analysis

Molecular Complex Detection (MCODE) is based on complex algorithms that cluster objects with similar properties. We clustered the data in the potential target PPIs network of the SHXP in the treatment of COVID-19.^[33–35]^ The Cytoscape plugin MCODE was applied to analyze clustering modules in the PPIs network. Besides, KEGG enrichment analysis was conducted on each module through R language (Cluster Profiler package), and the threshold value was set with *P* ≤ .05 as a screening standard. The results were visualized and output in bar graphs and bubble graphs.

### 2.8 SHXP anti-COVID-19 prescription validation

TCM Anti COVID-19 (TCMATCOV, http://tcmatcov.bbtcml.com) is a platform to predict the efficacy of the anti-coronavirus pneumonia effect of TCM. TCMATCOV is based on the interaction network imitating the disease network of COVID-19.^[36]^ TCMATCOV utilizes a quantitative evaluation algorithm to analyze disease network disturbance after multi-target drug attacks to predict potential drug effects. The formula composition of SHXP was input into TCMATCOV V1.0 with pinyin form, and the confidence score of protein interaction was set as 0.5 to analyze the robustness of the COVID-19 disease network. The negative control group selected Banxia Tianma Baizhu decoction, while the positive control group selected Qingfei Paidu decoction. This platform uses four kinds of topology measurement values to measure network stability, including average degree, average shortest path, degree centrality, and closeness centrality. The total disturbance score is used as a comprehensive indicator to assess the degree of drug interference with the COVID-19 disease network's robustness.

### 2.9 Molecular docking simulation

The Research Collaboratory for Structural Bioinformatics Protein Data Bank (RCSB PDB, https://www.rcsb.org), the US data center for the global PDB archive, serves thousands of data depositors in the Americas and Oceania and makes available 3D macromolecular structure data free of charge. The RCSB PDB database was used to retrieve and download the PDB ID of the potential target proteins, whereas the structure of active compounds was downloaded in the traditional Chinese medicine systems pharmacology database and analysis platform database as a ∗mol 2 format. The compound ligand and protein were converted to the “PDBQT” format by AutoDock software. Grid Box was set with the appropriate size and location. Finally, AutoDock Vina was performed for molecular docking. Generally, the binding energy ≤−5.0 kj/mol can be considered an excellent binding effect. Pymol software was used to visualize molecular docking results.^[37–39]^

## 3 Results

### 3.1 Compound-putative target network

**Figure 2:**
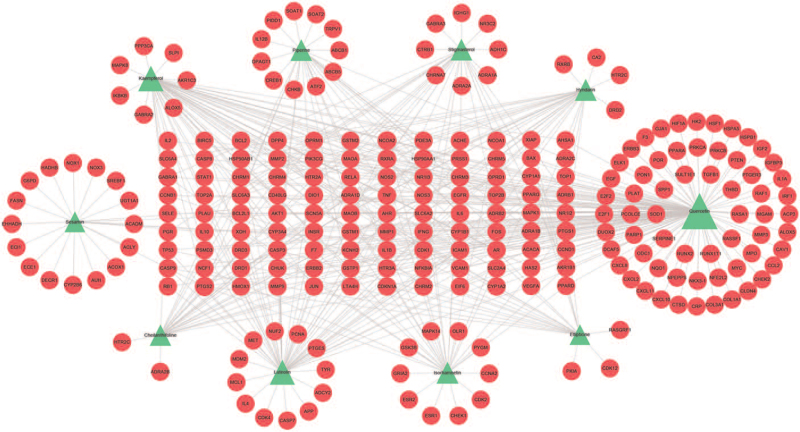
Compound-putative target network of SHXP. The green color indicates the chemical composition; the red color indicates the targets.

A total of 92 compounds from SHXP were screened. Of these compounds, 4 were from Seheixang, 4 from Anxixiang, 11from Chenxiang, 7 from Tanxiang, 8 from Dingxiang, 18 from Xiangfu, 24 from Muxiang, 9 from Ruxiang, 13 from Biba. After removing duplicated targets, all active compounds had 279 targets. The compound and target data were introduced into Cytoscape to gain network topology analysis data. In the network, the node's degree indicates the number of routes it takes to connect to other nodes. According to the network's topological properties, nodes with a more considerable degree would be screened for further analysis. We obtained the top 10 degree compounds by analyzing the network topology data, including quercetin (degree = 120), kaempferol, luteolin, isorhamnetin, hyndarin, piperine, stigmasterol, cheilanthifoline, sesamin, ellipticine, seeing Table 1 for details. Then, we used Cytoscape to construct a critical compound–target network that comprised 260 nodes (10 compound nodes and 250 target nodes) and 457 edges (Fig. 2). The green nodes represent the compound molecules, and the red nodes represent the compound targets. Each edge represents the interaction between the compound and its target. In this network, there is a phenomenon in which a compound corresponds to multiple targets, and several compounds share a common target simultaneously, which reflects that the mechanisms of SHXP in treating COVID-19 are composed of multiple components and targets, which is following the characteristics of many TCM prescriptions and drugs that have a curative effect.

**Table 1:**
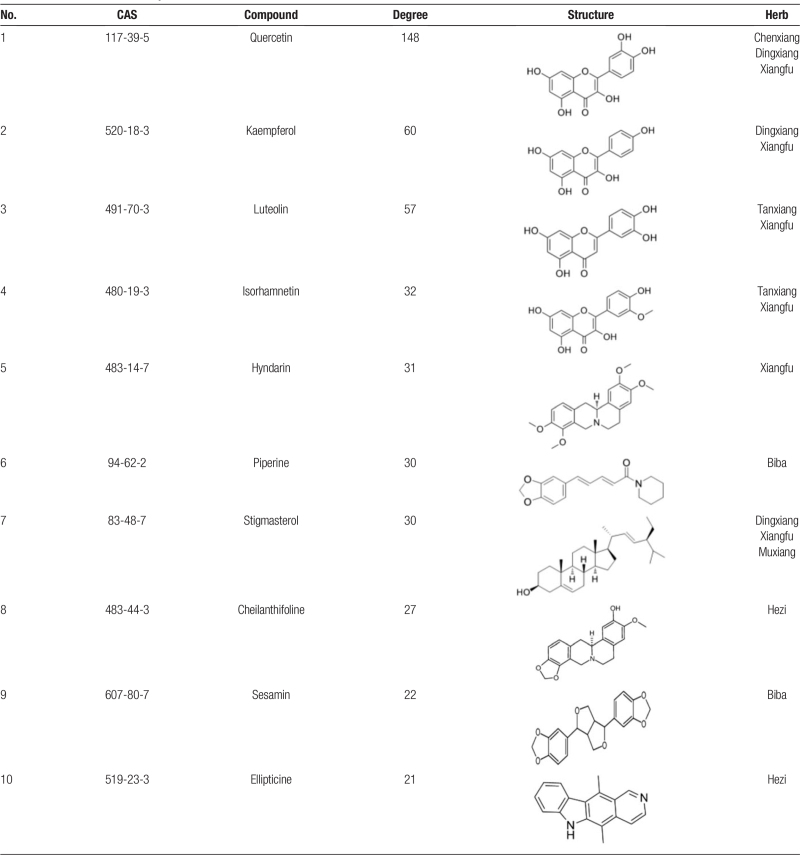
Information on active components.

### 3.2 COVID-19 targets

COVID-19 disease targeted genes/proteins were mined by GeneCards and OMIM databases. In GeneCards database, we obtained 324 COVID-19 related genes with relevance scores higher than zero, and among which 20 targets that scored higher than 2 were considered as essential COVID-19 targets, namely angiotensin-converting enzyme II (ACE2), dipeptidyl-peptidase 4, interleukin 2 receptor, alpha, cathepsin L, interleukin 6 (IL6), interleukin 10 (IL10), angiotensin-converting enzyme (ACE), vascular endothelial growth factor A (VEGFA). In the OMIM database, we gained 2 related genes of COVID-19.

### 3.3 PPIs network and enrichment analysis of potential targets

**Figure 3:**
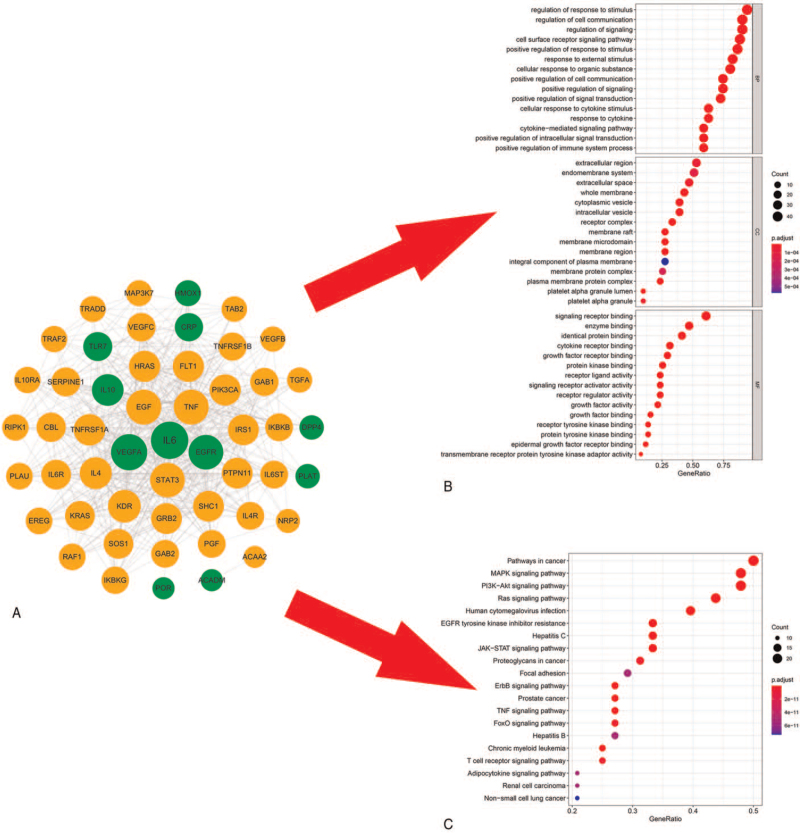
(A) PPIs network related to potential targets. The green color indicates primary proteins; the orange color indicates secondary proteins; the target's size is proportional to the degree value. (B) GO enrichment analysis of potential target PPIs network. *P* value <.01 and *q*-value <0.05. (C) KEGG enrichment analysis of potential target PPIs network. *P* value <.05 and *q*-value <0.05.

By intersecting the data of targets of compound and COVID-19, 11 SHXP/COVID-19 putative therapeutic targets were obtained. These targets were then brought in the STRING database to set up the PPIs network (Fig. 3A). The network had 51 nodes, with eleven primary targets represented in green (IL6, IL10, VEGFA, epidermal growth factor receptor [EGFR], dipeptidyl-peptidase 4, plasminogen activator, tissue, acyl-CoA dehydrogenase medium chain, cytochrome P450 Oxidoreductase [POR], Toll-like receptor 7, C-reactive protein, pentraxin-related, Heme Oxygenase 1) and 40 secondary targets in yellow, which interacted with 470 edges. Furthermore, to elucidate these genes’ biological functions, we analyzed the 51 targets by performing a GO enrichment analysis, and the top 45 significant GO entries (*P* < .05) were chosen according to the *P* value, as shown in (Fig. 3B). The results showed that the major genes were significantly involved in positive regulation of immune system process, regulation of cell communication, regulation of response to the stimulus, regulation of signaling, cellular response to cytokine stimulus, response to an external stimulus, and response to cytokine. The results of GO analyses showed that essential genes exist in extracellular space, endomembrane system, cytokine receptor binding, growth factor activity, receptor tyrosine kinase binding, enzyme binding, identical protein binding, growth factor binding, whose functions at the molecular level mainly included regulating the immune system and the production of cytokines. Thus, we speculated that SHXP exerted its pharmacological effects on COVID-19 by simultaneously involving these biological processes and molecular functions. By searching the Metascape database, 122 KEGG pathways with *P* values ≤.05 were also collected and analyzed. The top twenty crucially significant pathways were selected for further study and are displayed in the advanced bubble diagram (Fig. 3C). These pathways were associated with the Janus kinase (JAK)-signal transducer and activator of transcription (STAT) signaling pathway (hsa04630), phosphatidylinositol 3 - kinase (PI3K)-Protein kinase B (Akt) signaling pathway (hsa04151), ErbB signaling pathway (hsa04012), T cell receptor signaling pathway (hsa04660), tumor necrosis factor (TNF) signaling pathway (hsa04668), Human cytomegalovirus infection (hsa05163), FoxO signaling pathway (hsa04068), Chronic myeloid leukemia (hsa05220), proteoglycans in cancer (hsa05205), hepatitis B (hsa05161), advanced glycation end products-receptor for advanced glycation end products signaling pathway in diabetic complications (hsa04933), apoptosis (hsa04210), nuclear factor kappa-B signaling pathway (hsa04064), hypoxia-inducible factor 1 (HIF-1) signaling pathway (hsa04066), interleukin 17 (IL-17) signaling pathway (hsa04657), Toll-like receptor (TLR) signaling pathway (hsa04620), B cell receptor signaling pathway (hsa04662), Chemokine signaling pathway (hsa04062), Th17 cell differentiation (hsa04659), Influenza A (hsa05164). Detailed information about the top twenty significant pathways was supplemented in Figure 3.

### 3.4 Module analysis

**Figure 4:**
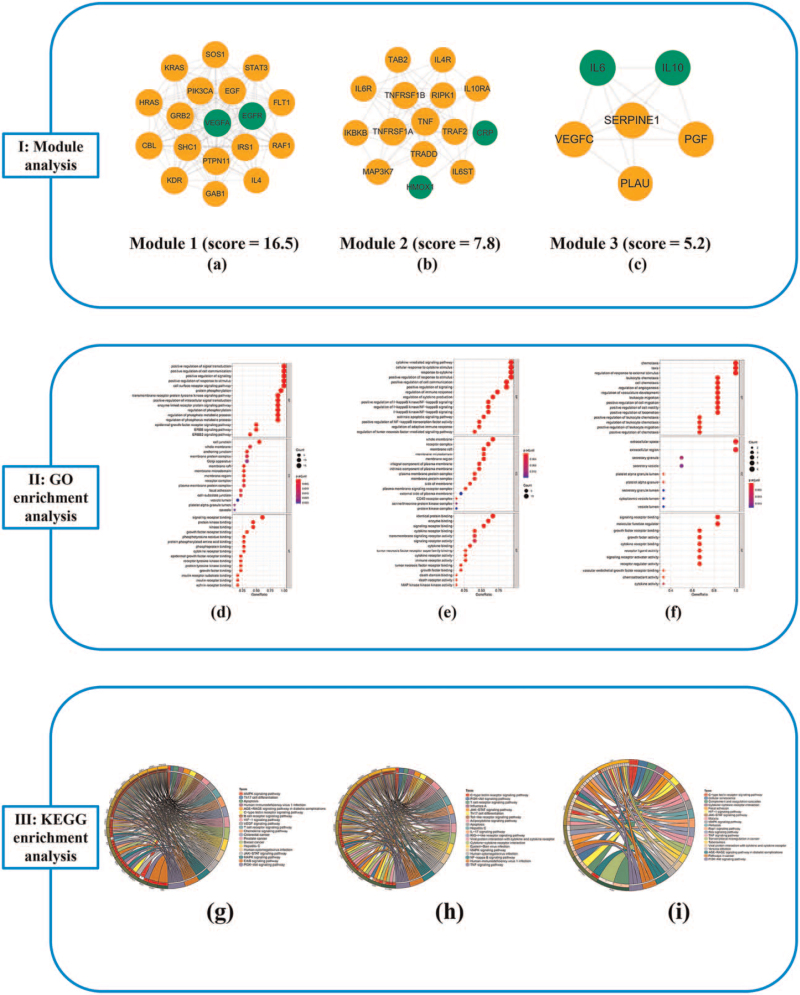
Analysis of clusters and functional enrichment analysis. I: Clusters of the potential PPIs network. (A) Module 1 (score = 16.5). (B) Module 2 (score = 7.8). (C) Module 3 (score = 5.2). Green circles represent intersection targets of SHXP and COVID-19, and the orange circles represent the secondary targets of intersecting targets. II: GO enrichment analysis for each cluster. The top 45 GO terms were shown in the figure; *P* value <.05 and *q*-value <0.05. (d) GO enrichment analysis of Module 1. (E) GO enrichment analysis of Module 2. (F) GO enrichment analysis of Module 3. III: KEGG pathway enrichment analysis for each cluster. *P* value <.05 and *q*-value < 0.05. (G): Pathway analysis of Module 1. (H) Pathway analysis of Module 2. (I) Pathway analysis of Module 3. The top 20 KEGG pathways were shown in the figure. The y-axis shows significantly enriched KEGG pathways, and the x-axis shows the gene counts.

Because clustering modules might represent some pivotal characteristics of PPIs networks and probably contain specific biological significance, we analyzed the PPIs network of targets for SHXP against COVID-19 by using MCODE and detected the 3 modules (Fig. 4(I)). The scores of the 3 modules are all greater than 5. Module 1 (scores = 16.5) contains 18 nodes and 141 edges; Module 2 (scores = 7.8) includes 18 nodes and 141 edges; and Module 3 (scores = 5.2) involves 6 nodes and 13 edges (Fig. 4A). Then, we performed GO enrichment analysis (*P* value <.05) of the 3 modules to gain insights into the cellular component, molecular function, and biological processes that are affected in COVID-19. The results indicated that Module 1 was highly correlated with cell transduction, enzyme activity, immunologic process, and metabolic process. Module 2 was highly associated with cytokine production, apoptosis, and immune regulation. Module 3 was highly linked to cell movement, angiogenesis, chemokine production, and immune system regulation. Overall, the potential targets were highly connected with cytokines and the immune system. Subsequently, we performed KEGG enrichment analysis (*P* value <.05) of the 3 modules to obtain essential pathways of SHXP in treating COVID-19. Enrichment analysis of the KEGG pathway showed that Module 1 screened 100 pathways, mainly related to signal transduction pathways and inflammatory pathways. A total of 55 pathways were screened in Module 2, related to inflammation, immune pathways, and viral infection pathways. Module 3 has 27 pathways, mainly related to cytokine, immune, and cancer signaling pathways. Further analysis shows that the three motabledules contain 3 identical pathways: the JAK-STAT, PI3K-Akt, and C-type lectin receptor signaling pathways, suggesting these 3 pathways may be key pathways SHXP against COVID-19. GO enrichment analysis results were visualized using the R package, and the top twenty pathways of each module were selected for ribbon visualization (Fig. 4(II) and Fig. 4(III)).

### 3.5 Hub genes of PPIs network

**Figure 5:**
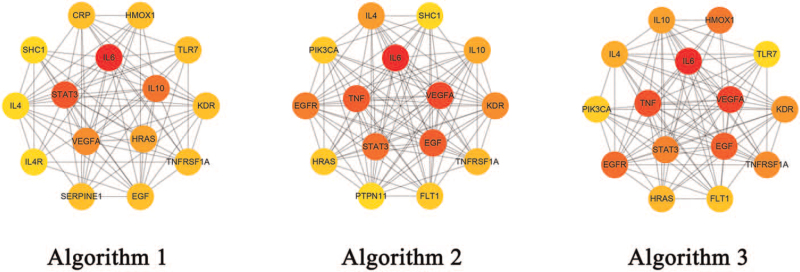
Hub genes under different algorithms. (A) Algorithm of bottleNeck. (B) Algorithm of closeness. (C) Algorithm of betweenness.

We use 3 algorithms to look for hub genes of the PPIs network of SHXP/COVID-19 intersection targets. There were 3 genes, IL6, IL10, and STAT3, with a score of ≥5 by using the BottleNeck algorithm analysis (Fig. 1). Besides, IL6, VEGFA, epidermal growth factor (EGF), TNF, and STAT3 have higher scores, all of which are greater than 40 with the Closeness algorithm, and they may be considered as essential genes. While applying the Betweenness algorithm, IL6, VEGFA, TNF, and EGF scores are all over 100, suggesting that they may be critical genes in the network. Through the comprehensive analysis of 3 algorithms, we can find that the hub genes in the network are IL6, IL10, VEGFA, EGF, TNF, and STAT3. These may be critical genes that play a vital role in the pathway, implicating that we should focus on their respective pathways and conduct a detailed analysis. The hub genes were mostly enriched in JAK-STAT, PI3K-Akt, C-type lectin receptor, TNF, and IL-17 signaling pathways, which suggested that the compounds in SHXP may treat COVID-19 through the above pathways by acting on related targets (Fig. 5).

### 3.6 Prediction of SHXW-COVID-19 disease treatment by TCMATCOV

TCMATCOV platform adopts 4 indexes: average shortest path, average degree, degree centrality, and closeness centrality, to evaluate disease networks’ robustness together. The shorter the average path length, the greater the average degree, degree centrality, and closeness centrality are the greater the network disturbance to disease. The total disturbance score evaluates the disturbance effect of drugs on the disease network from the overall level; the higher the value, the higher the damage degree of drugs to the disease network's stability, the more significant treatment effect for COVID-19. The total disturbance score and 4 indexes of SHXP are shown in Table 2. The total disturbance score of SHXP was 20.74, which was higher than the positive control group. By comparing the 4 indicators and the total indicators with the positive control, it can be seen that SHXP Pill has a strong disturbing effect on the COVID-19 disease network and can play a practical therapeutic effect on severe COVID-19 patients.

**Table 2:** Traditional Chinese medicine with different groups for COVID-19 network score.

	Network topology characteristics				
Groups	Average degree	Average shortest path	Degree centrality	Closeness centrality	Disturbance score
Suhexiang pill	−3.78	8.93	−1.24	−6.79	20.74
Qingfei Paidu decoction	−2.84	8.30	−1.20	−6.76	19.10
Banxia Tianma Baizhu decoction	−1.84	3.53	−0.76	−6.46	12.59

### 3.7 Molecular docking

**Figure 6:**
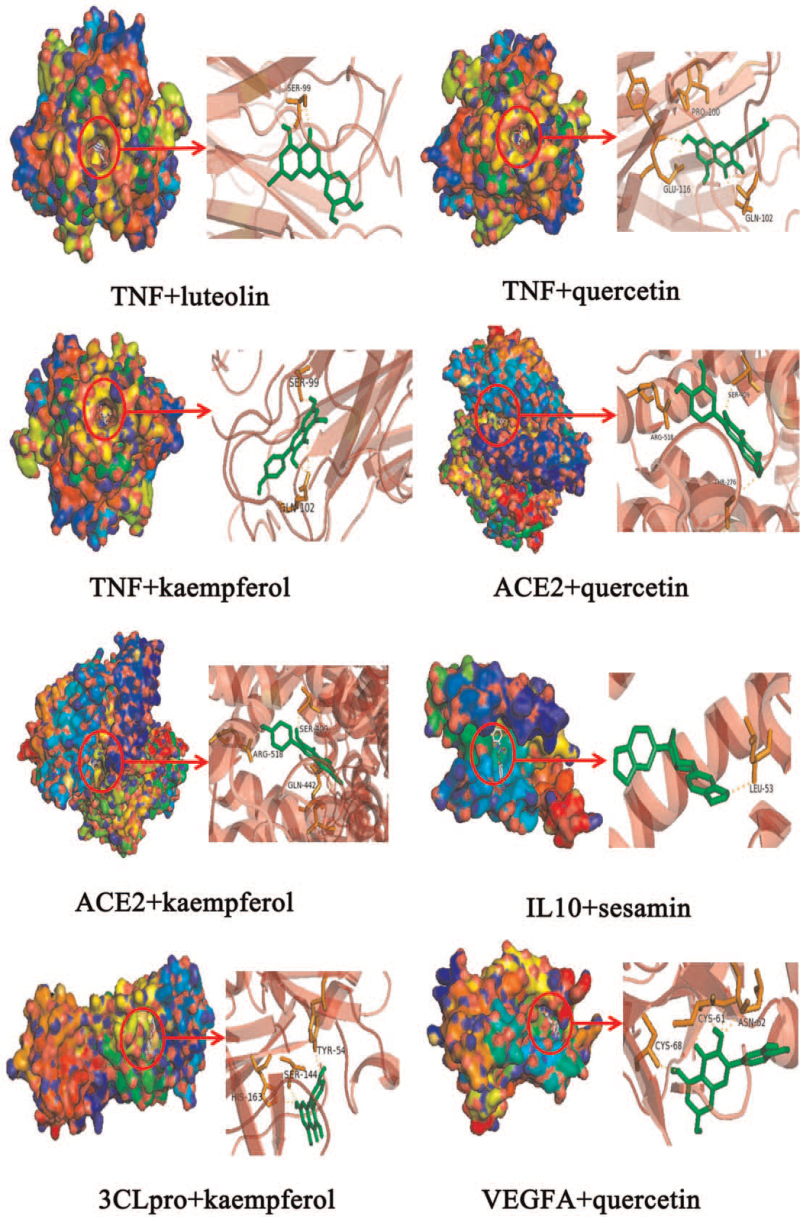
Detailed target-compound interactions with the 8 highest molecular docking affinities.

The screened core compounds and hub genes are selected for the molecular docking experiment. Simultaneously, since ACE2 and 3CL hydrolase (3CLpro) have been reported as potential targets for anti-COVID-19, these 2 proteins are also selected for molecular docking with 2 core compounds that have the highest degree. The docking details are shown in Table 3, and the binding energy values of key compounds in SHXP with their hub targets are all less than −6 kcal/mol, which suggests that the binding is significant; the less energy that is required, the more stable the binding. We selected the targets that bind to the most stable compound to display the 3D results, which included TNF-luteolin (affinity = −9.0 kcal/mol), TNF- quercetin (affinity = −8.9 kcal/mol), TNF-kaempferol (affinity = −8.9 kcal/mol), ACE2-quercetin (affinity = −8.2 kcal/mol), ACE2-kaempferol (affinity = −8.0 kcal/mol), IL10-sesamin (affinity = −8.2 kcal/mol), 3CLpro-kaempferol (affinity = −7.8 kcal/mol), and VEGFA-quercetin (affinity = −7.8 kcal/mol). In Figure 6, the red circle represents a small-molecule compound, and each small-molecule compound is bound to a large-molecule protein. The stick graph on the right describes the specific form of this interaction. The orange dashed line in the Figure 6 represents hydrogen bonding; there are fewer hydrophobic residues around the compound, and these residues are mainly bound to the target by electrostatic interactions (hydrogen bonding).

**Table 3:** Information on molecular docking.

NO.	Proteins	PDB ID	Test compounds	Affinity (kcal/mol)
1	IL6	4O9H	Quercetin	−6.9
2	IL6	4O9H	Piperine	−6.5
3	IL6	4O9H	Luteolin	−6.7
4	VEGFA	5DN2	Quercetin	−7.8
5	VEGFA	5DN2	Luteolin	−7.2
6	TNF	1DU3	Kaempferol	−8.9
7	TNF	1DU3	Quercetin	−8.9
8	TNF	1DU3	Luteolin	−9.0
9	TNF	1DU3	Piperine	−7.7
10	IL10	2H24	Quercetin	−6.4
11	IL10	2H24	Luteolin	−6.5
12	IL10	2H24	Sesamin	−7.9
13	ACE2	2AJF	Quercetin	−8.2
14	ACE2	2AJF	Kaempferol	−8.0
15	3CLpro	6LU7	Quercetin	−7.4
16	3CLpro	6LU7	Kaempferol	−7.8

### 3.8 Drug-compound-target-pathway network construction

**Figure 7:**
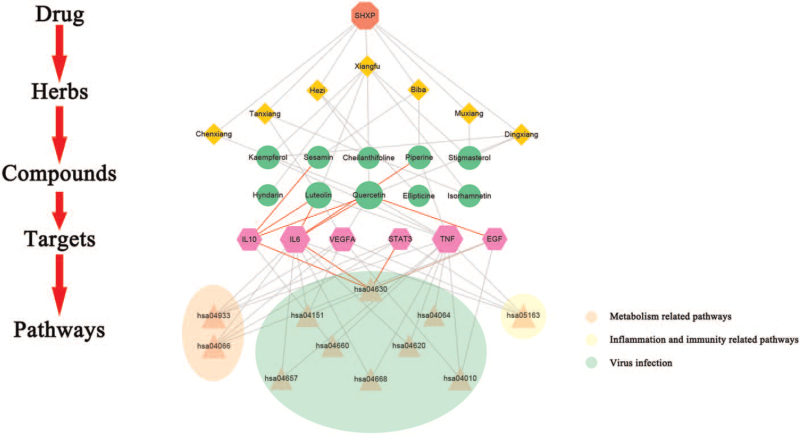
Herb-compound-target-pathway network. The red node represents the SHXP; the orange squares represent the herbs in the SHXP; the green circles represent each herb's ingredients; the pink hexagons represent the ingredients’ targets; and the triangular nodes represent the pathways enriched with the targets.

**Figure 8:**
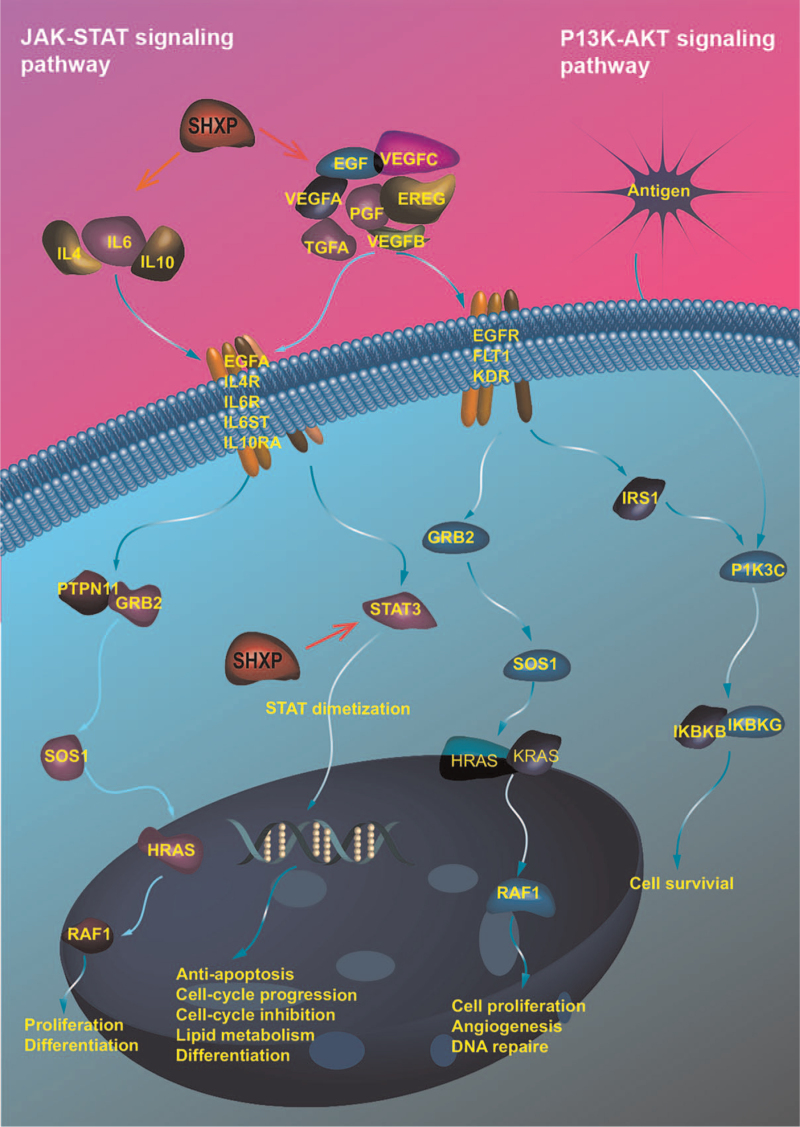
Illustration of the crucial signal pathway involving putative targets and known therapeutic targets of SHXP: JAK-STAT signaling pathway and PI3K-Akt signaling pathway pathways.

To systematically and holistically explain the interaction mechanisms among the different drugs, compounds, and main targets in SHXP, a “drug-core compounds-hub targets-pathway” network was established based on Cytoscape software. As shown in Figure 7, there were a total of 35 nodes and 68 edges. In the figure, the red squares represent the SHXP, the orange quadrilaterals represent the herbs in the SHXP, the green circles represent each herb's components, the pink hexagons represent the components’ targets, and the yellow triangles represent the pathways enriched in the targets. According to the PPIs network's path enrichment analysis and the 3 modules analysis results, we found that the JAK-STAT signaling pathway (hsa04630) and PI3K-Akt signaling pathway (hsa04151) pathway are the intersection modules of the 4 enrichment analyses. The pathways contain multiple hub genes, so we use the red line to highlight the 2 pathways. It is suggested that the 2 pathways may be the primary therapeutic pathways of SHXP. Importantly, by combining the obtained information of compound-target and hub genes, we found that IL6, IL10, VEGFA, EGF, TNF, and STAT3 were considered key targets because they also exist in 2 importa nt pathways related to inflammation (Fig. 8).

## 4 Discussion

In contrast to patients with mild and common types, patients with severe and critical cases of COVID-19 often develop dyspnea and or hypoxemia 1 week after the onset of the disease, and severe cases can rapidly develop into acute respiratory distress syndrome (ARDS).^[40]^ The expression levels of various pro-inflammatory factors in plasma were significantly higher in severe patients, and studies have suggested that “cytokine storm” is the primary pathogenic mechanism in severe patients with COVID-19. Cytokine storm, also known as an inflammatory storm, is an overactive immune response to external stimulus. In such an overactive immune response, cytokines and leukocytes in tissues and organs may increase uncontrollably due to a specific positive feedback regulatory mechanism.^[41,42]^ However, the molecular mechanisms underlying the effects of SHXP in the treatment of COVID-19 are not clear. Hence, in the present study, a set of network pharmacology methods were used to predict, elucidate and confirm the potential mechanisms of SHXP against COVID-19 by integrating target prediction, network construction, mechanism analysis, and molecular docking verification.

TCM has multi-targets and integral regulating effects. Some TCM components have specific antibacterial and antiviral effects. Their antiviral research pays more attention to the overall effect, explaining from a specific index and considering the interaction between various indexes or factors. The overall regulation effect helps prevent cytokine storm from the source.^[43]^ Cytokines include pro-inflammatory cytokines such as interleukin -6 (IL-6), IL-2, tumor necrosis factor-α (TNF-α) and anti-inflammatory cytokines such as interleukin-10 and IL-1. The balance between the 2 is a critical factor in maintaining normal immune function that resists disease. Cytokines interact with each other in the body and are jointly involved in the inflammatory response, which has antagonistic and promoting effects and forms a complex immune regulatory network.

Our study screened out 5 main active components of SHXP, named quercetin, kaempferol, luteolin, piperine, and sesamin. They are core compounds with anti-COVID-19 effects after screening through the compound-target network and molecular docking verification. Quercetin has anti-inflammatory, antibacterial, antiviral, gastric protection, and immune regulatory functions.^[44]^ Studies have shown that quercetin can reduce the expression of transforming growth factor, beta 1, actin alpha 2, smooth muscle, and TNF-α, inhibit rat alveolar cell apoptosis, and reduce rat lung tissue inflammation and fibrosis damage.^[45]^ At the same time, it can reduce lung inflammation by inhibiting the activation of the JAK2/STAT3 signaling pathway. Quercetin can also inhibit the activation of signal transducer and activator of transcription factor NF-кB, inhibit the production of TNF-α and nitrous oxide by macrophages, microglia, and mast cells, reducing the fatal shock response.^[46]^ Luteolin has been found to have anti-inflammatory, anti-viral, and other pharmacological effects, and to exhibit lung-protective effects, which may be related to its inhibition of the release of IL-6 and TNF-α.^[47]^ Kaempferol can prevent the phosphorylation of PI3K and Akt from reducing the inflammatory reaction after influenza virus infection, inhibit the release of inflammatory factors such as IL-6 and TNF-α and reduce the damage of oxidative stress to the lung.^[48–50]^ Pathak and colleagues found that piperine has an immunomodulatory effect and can improve cell apoptosis and immune function suppression in mice.^[51,52]^ Piperine can regulate oxidative stress and apoptosis and relieve the inhibition of cell proliferation, thereby regulating the morphological changes and cytokine release of T cells and B cells. Besides, piperine can play an anti-inflammatory role by inhibiting the expression of endothelial cell adhesion molecules that TNF-α induced.^[54]^ The mechanism involved may be due to the fact that it interferes with the early signal transduction of TNF-α, thereby affecting the activation of nuclear factor kappa-B. Sesamin can significantly reduce IL-6 and IL-1 mRNA expression in human primary synovial fibroblasts, indicating that sesamin can block TNF-α induced pro-inflammatory cytokine mRNA expression.^[53]^

Through 3 algorithms of hub gene screening and molecular docking verification, we obtained 4 potential therapeutic targets of SHXP, which are IL6, IL10, VEGFA, and TNF. Lipopolysaccharide (LPS) can stimulate the synthesis and secretion of various pro-inflammatory factors, including IL-6, by binding to TLR and other LPS receptors on cell membranes.^[54]^ IL-6 is a crucial inflammatory factor in the initial stage of inflammation. Studies have shown that quercetin can reduce inflammation by inhibiting the expression of IL-6mRNA in neutrophils during the process of LPS-induced production of inflammatory cytokine IL-6, thereby decreasing the synthesis and secretion of IL-6. IL10 is a protein-coding gene that can encode cytokine. This cytokine has pleiotropic effects in immunoregulation and inflammation, which has profound anti-inflammatory functions, limiting excessive tissue disruption caused by inflammation.^[55]^ Interleukin-10 can block NF-кB activity and regulate the JAK-STAT signaling pathway.^[56]^ NF-кB, a critical nuclear transcription factor, widely exists in eukaryotic cells, which regulates many molecules in various early immune response and inflammatory response stages. NF-кB plays an essential role in the occurrence and development of diseases involving inflammatory mediators, cytokines, and proteases. The TNF family refers to a group of cytokines that can cause cell death (apoptosis), including TNF-α and TNF-β. TNF-α is mainly produced by activated macrophages, which may be involved in systemic inflammation. TNF-β is secreted by lymphocytes and mediates many inflammatory, immunostimulatory, and antiviral responses.^[57]^ VEGFA is a member of the PDGF/VEGF growth factor family. VEGF levels increase in SARS-CoV-2 infection and promote inflammation by increasing angiotensin II (Ang II) levels.^[58]^ In turn, Ang II elevates VEGF, creating a vicious cycle in releasing inflammatory cytokines. When SARS-CoV-2 infects the human body, it causes the immune cells in the body to secrete a large number of inflammatory factors, forming a cytokine storm that attacks the immune organs in the human body, seriously affects the respiratory, circulatory, and other systems, and even causes the death of patients. If left uncontrolled, the cytokine storm can eventually lead to respiratory problems such as breathing difficulties and even death. Combined with clinical studies, the expression levels of inflammatory factors in some severe patients with COVID-19 were significantly higher than those in general type patients. The results showed that the intensity of expression of these inflammatory factors was related to the degree of infection. The useful components in SHXP may further improve the clinical symptoms of patients by acting on inflammatory factors.^[59,60]^

KEGG enrichment analysis showed that the key targets were mainly concentrated in TNF signaling pathway, PI3K-Akt signaling pathway, MAPK signaling pathway, HIF-1 signaling pathway, and NF-кB pathway. Four hub genes, IL6, IL10, STAT3, and EGF, are enriched in the JAK-STAT signaling pathway, closely related to macrophages’ inflammatory differentiation. Inflammatory factors can activate the corresponding JAK-STAT, play the function of signal transduction and transcriptional activation, and then affect the M1/M2 type differentiation and inflammatory direction of macrophages. Under the stimulation of pro-inflammatory factors, macrophages can release a series of cytokines that can further promote the macrophages’ recruitment and M1-type polarization, resulting in the self-amplification effect of the body's inflammatory response. Many studies have shown that the JAK-STAT signaling pathway may play a vital role in regulating macrophage inflammatory initiation.^[61]^ Because JAK-STAT may have been involved in the control process of the M2-type activation and anti-inflammatory differentiation of macrophages, the pathway may significantly affect the start of macrophages’ proliferation. M2-type macrophages can secrete anti-inflammatory factors such as IL4 and IL10, which act in the elimination of inflammation and wound healing in the body. IL10 can inhibit the expression of TNF-α induced by LPS and reduce the production of pro-inflammatory cytokines.^[62]^ The essential compounds in SHXP may inhibit the M1-type differentiation of macrophages by regulating the JAK-STAT signaling pathway, reduce the production of pro-inflammatory factors, meanwhile promote the M2-type differentiation of macrophages to increase the production of anti-inflammatory factors, thereby preventing COVID-19 patients from being hurt by the “inflammatory storm.” The JAK-STAT pathway can interact with TLR to regulate M1-type polarization and macrophages’ inflammatory response. The TLR is called the bridge, which connects innate immunity and acquired immunity, whose negative feedback mechanism exerts anti-infection immunity advantages. The PI3K-Akt signaling pathway regulates cell differentiation, proliferation, activation, and antiviral activity by expressing a series of protein factors. In recent years, many in vivo and in vitro studies have demonstrated that the PI3K-Akt signaling pathway generates a critical negative regulatory in the pulmonary inflammatory response. PI3K inhibitors can alleviate pulmonary edema in mice and significantly alleviate pulmonary inflammatory response, suggesting that the core compound in SHXP may alleviate pulmonary inflammation in patients with COVID-19 by controlling the PI3K-Akt signaling pathway.^[63]^ Besides, HIF-1 signaling pathway, TNF signaling pathway, IL-17 signaling pathway, T cell receptor signaling pathway and other signaling pathways are also enriched in hub genes, which may be related to the effect of SHXP on COVID-19. The HIF-1 signaling pathway is involved in hypoxia regulation in the human body and can activate the transcription of multiple genes under hypoxia conditions to increase oxygen delivery. Autopsy results showed that the COVID-19 patient had diffuse alveolar injury in both lungs with exudation of cellular mucus and detachment of alveolar epithelium, clinically presented with dyspnea, and eventually died of respiratory failure.^[64]^ Therefore, regulation of HIF-1 signaling pathway could reduce clinical hypoxia symptoms in patients with severe COVID-19. TNF signaling pathway was an essential pathway in the inflammatory response, in which related factor receptors can also induce apoptosis. Quercetin acted as an anti-inflammatory by regulating TNF signaling pathway.^[65–67]^

ACE is a receptor in the airway, alveoli, and vascular endothelium. COVID-19 uses ACE to enter type II pneumocytes cells to induce ACE2 internalization and shedding, resulting in the occurrence and development of ARDS. The combination of SARS-CoV-2 and ACE2 was the leading cause of COVID-19.^[68]^ SARS-CoV-2 3CL hydrolase is a vital protein of the virus itself, which can hydrolyze polyproteins into mature proteins with replication and transcription functions. It is required to realize RNA replication of the virus and is considered a useful target against the SARS-CoV-2 virus. According to the network diagram of “drug-ingredient-target,” quercetin and luteolin ranked the highest in the degree were selected for molecular docking with ACE2 target and SARS-CoV-2 3CL hydrolase, the result showed that all the key compounds in the network had a strong affinity with ACE2 protein and SARS-CoV-2 3CL protein.

Although our study explored the molecular mechanisms of SHXP, there are still some limitations. First, the data of the study were obtained from existing databases, so the authenticity and integrity of the result depend on the data. In addition, further experiments are needed to confirm the prediction results of SHXP because our study relied on data analysis.

## 5 Conclusions

In this study, we systematically analyzed the therapeutic targets, signaling pathways, the potential mechanisms by which SHXP is useful in treating COVID-19 based on network pharmacology, and molecular docking approaches. The therapeutic efficacy of SHXP against COVID-19 is likely mediated via the regulation of 4 targets, namely, IL6, IL10, VEGFA, and TNF. Furthermore, the molecular docking simulation demonstrated an excellent affinity of these 4 hub genes, as well as ACE2 and 3CLpro with quercetin, kaempferol, luteolin, and piperine in SHXP. KEGG enrichment analysis illustrated that SHXP may simultaneously act on various pathways, including JAK-STAT signaling pathway, PI3K-Akt signaling pathway, TNF signaling pathway, NF-кB signaling pathway, TLR signaling pathway, IL-17 signaling pathway, HIF-1 signaling pathway, and T cell receptor signaling pathway. Our study showed that SHXP, a TCM formula, might be useful in treating COVID-19 by mainly regulating the immune response, inhibiting the inflammatory storm, and inhibiting virus entry into cells replicating themselves. These findings may offer a reference basis for the clinical application and further mechanism exploration by which SHXP exerts effects against COVID-19.

## Author contributions

**Conceptualization:** Jialin Li, Peizhi Ye.

**Data curation:** Jialin Li, Yingying Tan, Chao Wu, Yingying Liu, Xiaotian Fan.

**Formal analysis:** Jialin Li, Yingying Tan, Bei Zhang.

**Funding acquisition:** Jialin Li, Jiarui Wu.

**Investigation:** Jialin Li, Jianping Yi, Yi Xu.

**Methodology:** Jialin Li, Shan Lu, Xinkui Liu.

**Project administration:** Xinkui Liu.

**Resources:** Jialin Li, Zhishan Wu.

**Software:** Jialin Li, Zhihong Huang, Xinkui Liu, Liangliang Shen.

**Supervision:** Peizhi Ye.

**Validation:** Peizhi Ye, Lu Yao.

**Visualization:** Zhihong Huang, Haojia Wang.

**Writing – original draft:** Jialin Li.

**Writing – review & editing:** Hua Luo, Antony Stalin, Yanfang Mou.
